# KBG syndrome

**DOI:** 10.1186/s13023-017-0736-8

**Published:** 2017-12-19

**Authors:** Dayna Morel Swols, Joseph Foster, Mustafa Tekin

**Affiliations:** 1 Division of Clinical and Translational Genetics, Dr. John T. Macdonald Foundation Department of Human Genetics, University of Miami Miller School of Medicine, 1501 NW 10th Avenue, BRB-336 (M-860), Miami, FL 33136 USA; 2 John P. Hussman Institute for Human Genomics, University of Miami Miller School of Medicine, Miami, FL USA; 3 Department of Graduate Education, School of Medicine, 130 Mason Farm Road, CB7108, Chapel Hill, 27599 USA

**Keywords:** ANKRD11, KBG syndrome, Macrodontia, Review, Short stature

## Abstract

**Clinical Description:**

KBG syndrome is characterized by macrodontia of upper central incisors, distinctive craniofacial features such as triangular face, prominent nasal bridge, thin upper lip and synophrys; skeletal findings including short stature, delayed bone age, and costovertebral anomalies; and developmental delay/intellectual disability sometimes associated with seizures and EEG abnormalities. The condition was named KBG syndrome after the initials of the last names of three original families reported in 1975.

**Epidemiology:**

The prevalence of KBG syndrome is not established. There are over 100 patients reported in the literature. It is likely that KBG syndrome is underreported due to incomplete recognition and very mild presentations of the disorder in some individuals. KBG syndrome is typically milder in females.

**Etiology:**

Causative variants in *ANKRD11* have been identified in affected individuals. The vast majority of identified variants are loss of function, which include nonsense and frameshift variants and larger deletions at 16q24.3. Haploinsufficiency appears to be the mechanism of pathogenicity.

**Genetic Counseling:**

Familial and de novo cases have been reported. Causative de novo variants occur approximately one third of the time. Transmission follows an autosomal dominant pattern. The syndrome displays inter- and intra-familial variability.

**Electronic supplementary material:**

The online version of this article (10.1186/s13023-017-0736-8) contains supplementary material, which is available to authorized users.

## KBG syndrome- key points summary

- KBG syndrome is a rare, pan ethnic, autosomal dominant disorder, typically with more severe findings in males
- Clinical presentation is characterized by macrodontia, distinctive craniofacial findings, skeletal findings, post-natal short stature, and developmental delays [1]
- Point mutations, indels, and large deletions in *ANKRD11* account for most but not in all cases [2–4]
- While there are no formal diagnostic criteria, several have been proposed. A clinical diagnosis of KBG syndrome should be suspected in an individual with two or more of the findings highlighted by an asterisk OR one finding highlighted by an asterisk with at least two more findings: [1–3, 5]
  - ▪ *Macrodontia of permanent upper central incisors
  - ▪ *Developmental delay or mild/moderate intellectual disability or learning difficulty associated with behavioral issues
  - ▪ *Characteristic facial appearance
  - ▪ *Post-natal short stature
  - ▪ *1st degree relative with KBG syndrome
  - ▪ Conductive hearing loss due to recurrent otitis media
  - ▪ Palatal abnormalities
  - ▪ Hair findings (eg. low hair line, coarse hair)
  - ▪ Delayed bone age (>2SD below mean)
  - ▪ Large anterior fontanelle with delayed closure
  - ▪ Hand findings
  - ▪ Costovertebral anomalies
  - ▪ Scoliosis
  - ▪ EEG abnormalities with or without seizures
  - ▪ Feeding difficulties
  - ▪ Cryptorchidism in males
- Individuals with 16q24.3 deletion involving the *ANKRD11* gene have similar findings to those with intragenic variants, but also have a higher incidence of other findings such as brain anomalies, congenital heart defects, severe astigmatism, thrombocytopenia, and potentially autism spectrum disorder among other behavioral problems [2, 6–8]
- Missense *ANKRD11* variants should be interpreted with caution as the vast majority of reported variants have been loss of function

## Background

KBG syndrome (Orpha 2332; MIM 148050) was first described in 1975 by Herrmann et al. [9] as a new “malformation/retardation syndrome” in three families. Developmental delays, short stature, dysmorphic features, and macrodontia were common findings among these families. The condition was named KBG syndrome after the initials of the last names of three original families [9]. While originally described as an autosomal dominant condition, subsequent reports showed that males were much more severely affected than females; hence for many years X-linked inheritance remained a possibility. Subsequent families supported autosomal dominant inheritance, which was confirmed by demonstration of causative *ANKRD11* variants in affected individuals [4]. It remains still unknown why females are less severely affected.

### Epidemiology

There have been over 100 cases of KBG syndrome reported [2, 3]. KBG syndrome was initially thought to be quite rare, however is likely underdiagnosed due to mild features [4]. Features are typically present at birth but may be difficult to recognize until developmental delays are apparent, or permanent teeth erupt. Also, it is likely that this syndrome is less frequently diagnosed since features are not severe and fairly common among other disorders [4]. The syndrome is pan-ethnic.

### Clinical findings and natural history

**Fig. 1:**
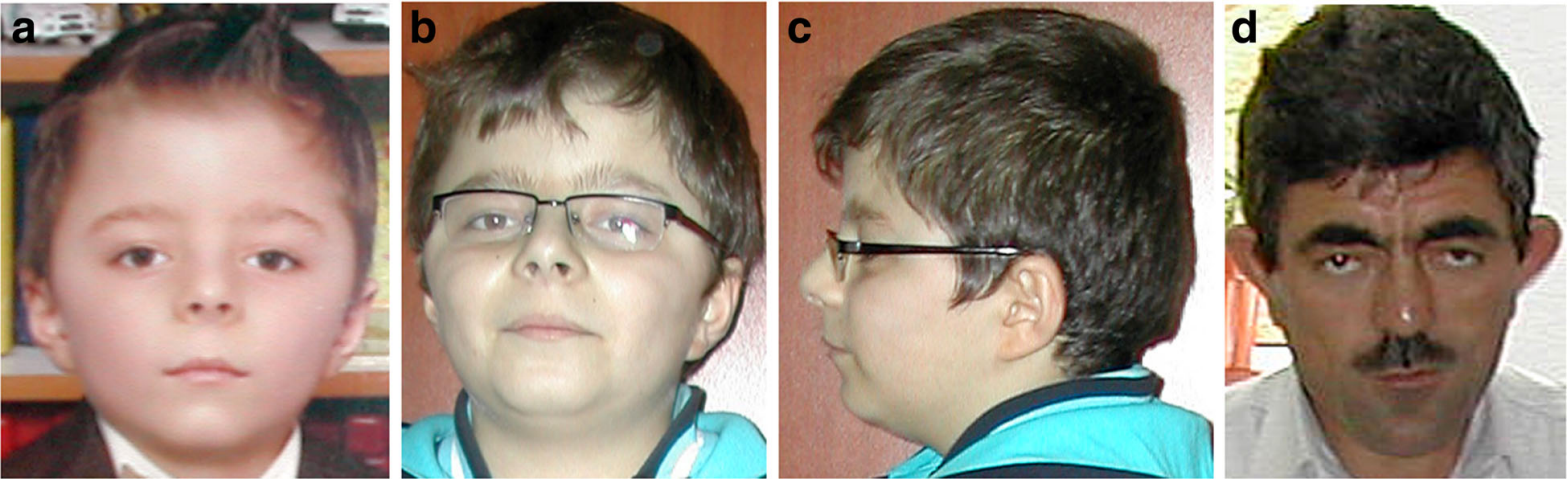
Craniofacial findings of KBG syndrome. (**a**, **b**, **c**) The same patient when he was 6 and 11 years old; (**d**) a 48-year-old patient. Note triangular face, synophrys, downslanting palpebral fissures, ptosis, high nasal bridge, anteverted nostrils, long philtrum, thin upper lip, and low anterior hairline

Following summary shows typical findings of KBG syndrome:

*Craniofacial findings (60–80% of reported individuals):* Triangular face, brachycephaly, synophrys and hypertelorism. A prominent high nasal bridge, anteverted nostrils, long philtrum, and thin upper lip are also common [2, 10] (Fig. 1).

**Fig. 2:**
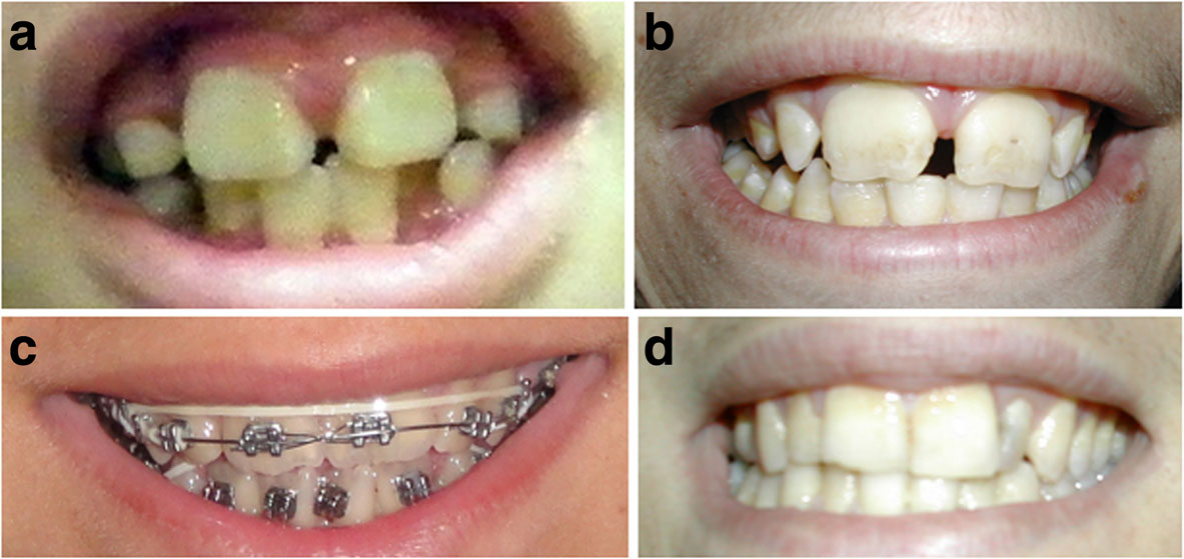
Macrodontia of permanent upper central incisors (**a**, **b**) Typical appearance of central upper incisors; (**c**) Prominent mamelons; (**d**) Slightly increased width of upper central incisors

*Dental findings (85% of reported individuals):* Macrodontia, especially of the permanent maxillary central incisors, is a main finding and is present in most but not all affected individuals [11]. This is typically defined as greater than or equal to 10 mm in males and 9.7 mm in females [1, 4]. Cleft teeth, shovel shaped incisors, enamel hypoplasia, oligodontia, dental pits and supernumerary mamelons (rounded protuberances present on the cutting edge of an incisor tooth) can also be seen [11] (Fig. 2).

**Fig. 3:**
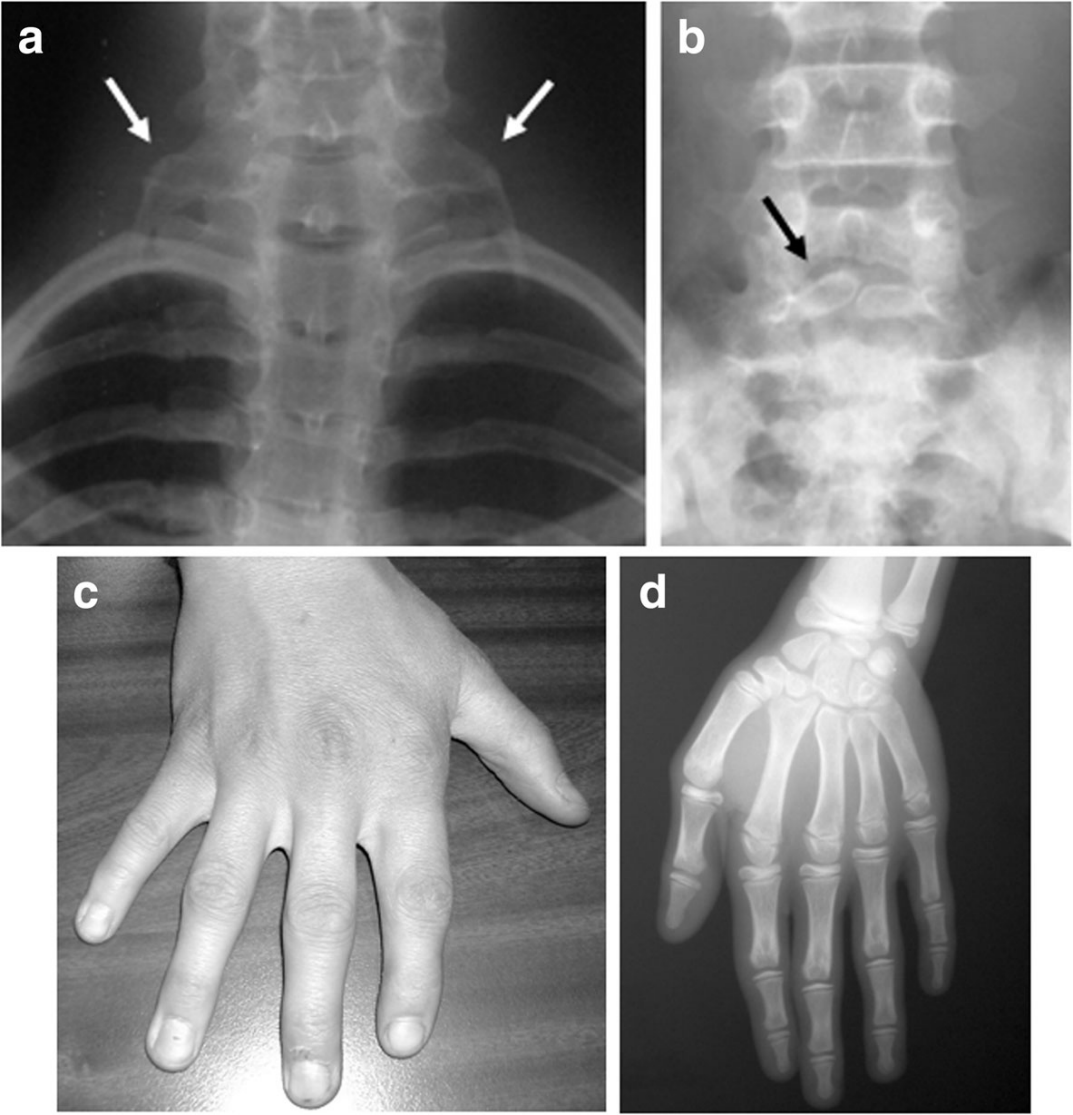
Skeletal findings. (**a**) Accessory ribs; (**b**) Spina bifida occulta; (**c**, **d**):Clinodactyly

*Skeletal findings (75% of reported individuals)*: Skeletal anomalies are common and variable among affected individuals. Most frequent are costovertebral anomalies [1]. Short stature (below −1 SD) is also seen in over half of patients with KBG syndrome [2]. Delayed bone age is an additional finding [5]. A large anterior fontanelle with delayed closure can also be seen [3]. Other abnormalities may include abnormal ribs, brachydactyly, clinodactyly, kyphosis, scoliosis, hip dysplasia, sternum abnormalities, and Wormian bones in skull [10]. (Fig. 3).

#### Neurological findings

*Intellectual abilities in childhood (93% of reported individuals have had developmental delays):* Cognitive skills can be quite variable among individuals. Most patients will have some degree of developmental delay, especially in speech [12]. There have not been any reports of regression. Average age for walking was 21 months [3, 10]. Average age for first words was 36 months [10]. Some children attend mainstream classes while others require special education [3].

*Intellectual abilities in adulthood:* It is not uncommon for verbal IQ to surpass performance IQ. Disability levels can range from mild learning disabilities, especially in females, to moderate intellectual disability, especially in males [12]. Completing a regular high school without additional support appears to be rare; however some reported adults have completed a trade school. More than half had jobs and were self-sufficient [2, 3].

*Seizures (up to 50% of reported individuals):* EEG abnormalities, with or without seizures have been reported in patients. Age of onset can be from infancy to the teens [3]. The type of epilepsy is nonspecific, although tonic-clonic seizures are most common. Treatment with antiepileptic drugs has proven to be effective in the majority of cases. Many patients have remission of symptoms after adolescence [12].

*Brain malformations:* Some brain abnormalities have been reported, although these are variable and are not included as part of the suggested diagnostic criteria. These abnormalities include cerebellar vermis hypoplasia [13], enlarged cysterna magna, Chiari I malformation, meningomyelocele [10, 14], and periventricular nodular heterotopia [15]. The frequency of brain anomalies has not been established in big cohorts.

*Psychiatric (50–70% of reported individuals):* Behavioral issues are common among patients with KBG syndrome although they can be variable. Milder problems can include poor concentration and restless movement. More severe problems can include obsessions, and deteriorating behavior when routines are changed. Anxiety and shyness are not uncommon, as are reports of difficulty in understanding social situations. [3]. While behavior issues are common among these individuals, autism spectrum disorder has been reported in varying frequencies suggesting ascertainment bias [2].

*Hearing (25–31% of reported individuals have had hearing loss):* Recurrent otitis media has been shown to cause hearing loss in some patients with KBG. All types of hearing loss, conductive, mixed and sensorineural, have been reported in association with the disease, with conductive loss to be the most common. Other otolaryngolic issues, such as soft cleft palate, bifid uvula, and velopharyngeal insufficiency have been reported [2, 10].

*Gastrointestinal findings:* Feeding difficulties, gastroesophageal reflux disease, and constipation have all been reported among patients with KBG syndrome [3].

*Other findings:* Undescended testicles have been reported in 25–35% of males [10]. Various ocular findings have been reported with KBG syndrome. While they are not common they include strabismus, congenital bilateral cataract, high myopia, and megalocornea [10]. Advanced puberty, some requiring treatment, has been reported in some individuals. Cardiac defects have been reported in approximately 10–26% of individuals, including atrial septal defects (ASD) and ventricular septal defects (VSD) among others [2]. Hypertrichosis has also been reported [3].

### Diagnosis of KBG syndrome

A clinical diagnosis of KBG syndrome should be suspected in an individual with two or more of the findings highlighted by an asterisk OR one finding highlighted by an asterisk with at least two more findings: [1–3, 5]

- ▪ *Macrodontia of permanent upper central incisors
- ▪ *Developmental delay or mild/moderate intellectual disability or learning difficulty associated with behavioral issues
- ▪ *Characteristic facial appearance
- ▪ *Post-natal short stature
- ▪ *1st degree relative with KBG syndrome
- ▪ Conductive hearing loss due to recurrent otitis media
- ▪ Palatal abnormalities
- ▪ Hair findings (eg. low hairline, coarse hair)
- ▪ Delayed bone age (>2SD below mean)
- ▪ Large anterior fontanelle with delayed closure
- ▪ Hand findings
- ▪ Costovertebral anomalies
- ▪ Scoliosis
- ▪ EEG abnormalities with or without seizures
- ▪ Feeding difficulties
- ▪ Cryptorchidism in males

A pathogenic or likely pathogenic DNA variant involving the *ANKRD11* gene confirms the diagnosis. Absence of an *ANKRD11* mutation does not exclude the diagnosis of KBG syndrome.

### Etiology

Single nucleotide variants and small indels within *ANKRD11* account for approximately 83% of pathogenic variants and larger copy number variants (mostly deletions) involving *ANKRD11* detectable by chromosomal microarray account for approximately 17% [2, 3].

There are currently no definitive genotype-phenotype correlations. Preliminary evidence suggests that individuals with a microdeletion involving only *ANKRD11* tend to have less neurological involvement than intragenic mutation carriers [16]. Individuals with a larger deletion, encompassing *ANKRD11* and other surrounding genes appear to have increased risk of neurobehavioral abnormalities [2, 6–8, 16]. It has also been seen that individuals with microdeletions have a higher incidence of congenital heart defects, astigmatism, and thrombocytopenia than those with intragenic mutations [16].

The syndrome is thought to be fully penetrant with variable expressivity. Males tend to be more severely affected than females for unknown reasons [4]. Intra-familial variability is commonly reported [3].

### Differential diagnosis

While many features are present from birth, it may be difficult to diagnose due to the mild or vague features that overlap with other syndromes [17]. Mild Cornelia de Lange syndrome (CdLS) has many similar findings including the facial features, developmental delay, growth retardation, hearing loss, and cryptorchidism. However, individuals with CdLS tend to have smaller head circumference and greater degrees of intellectual disability [18]. Russell Silver syndrome (RSS) also has overlap with KBG syndrome, exhibiting similar facial features, developmental delay, growth retardation, and cryptorchidism. However, individuals with RSS typically have prenatal onset growth retardation and limb asymmetry [19]. Aarskog syndrome has similar features to KBG syndrome including short stature, facial features, macrodontia, brachydactyly, vertebral anomalies, and cryptorchidism. However these individuals typically have a normal IQ and a characteristic shawl scrotum [20].

### *ANKRD11* molecular characterization

**Fig. 4:**
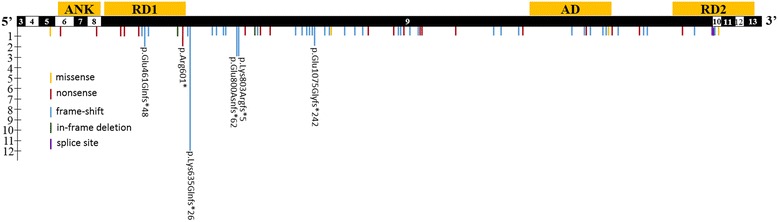
Location of reported causative variants in *ANKRD11*. Asterisks show location of recurrent variants, and y-axis gives reference for number of occurrences. From left to right these recurrent variants are as follows: NM_013275.5:c.1381_1384del (p.Glu461Glnfs*48), NM_013275.5:c.1801C > T (p.Arg601*), NM_013275.5:c.1903_1907del (p.Lys635Glnfs*26), NM_013275.5:c.2398_2401del (p.Glu800Asnfs*62), NM_013275.5:c.2408_2412del (p.Lys803Argfs*5), and NM_013275.5:c.3224_3227del (p.Glu1075Glyfs*242). The length of each line is proportional to the number of occurrences. ANK: Ankyrin repeat-containing domain (p.152–304). RD1: Repression Domain1 (p.318–611). Activation Domain (p.1851–2145). RD2: Repression Domain2 (p.2369–2663). Boundaries of exons are shown as alternating black and white boxes

Intragenic variants and microdeletions including *ANKRD11* have been reported to result in KBG syndrome [4, 8]. Seventy-nine of the 86 previously reported *ANKRD11* variants in patients with KBG syndrome are truncating and the majority of these cluster to the ninth exon (NM_013275.5), with only a handful of recurrent mutations being reported to date (Fig. 4, Additional file 1: Table S1). Only four missense *ANKRD11* variants have been reported to cause KBG syndrome (Fig. 4, Additional file 1: Table S1). However, phenotype was either mild or absent in some heterozygotes with missense variants; missense variants should be interpreted with caution. Confirming causality of rare missense variants requires multiple unrelated affected individuals with the same variant or co-segregation of variants with the phenotype in familial cases. A de novo missense variant in a person with high clinical suspicion supports the diagnosis. The prevalence of truncating variants points towards haploinsufficiency as the underlying cause of disease [2, 4]. This mechanism is supported by reports of microdeletion resulting in the complete loss of the coding region of *ANKRD11* [2]. However, a dominant-negative mechanism of action may be superseding haploinsufficiency in patients who carry a variant that leaves the N-terminus of the protein intact [21].

*ANKRD11* is located on the long arm of chromosome 16 and encodes for the protein Ankyrin repeat domain-containing protein 11. ANKRD11 contains four domains: an ankyrin repeat domain, a transcription activation domain, and two transcription repressions domains [22, 23] (Fig.4). Previous reports show that ANKRD11 primarily localizes to the nucleus [21]. ANKRD11 interacts directly with tumor suppressor protein TP53, and has been shown to fluctuate in abundance during the progression of the cell cycle [21, 22]. It also acts as a co-regulator. ANKRD11 interacts with p160 coactivators and several HDAC corepressors, which results in the inhibition of ligand-dependent transcriptional activation [23, 24]. More recently, ANKRD11 has been shown to regulate neural development by directing histone acetylation and gene expression [25].

### Mouse model

The mouse ortholog Ankrd11 has a 79% identity at the amino acid level with human ANKRD11. A chemically induced (N-ethyl-N-nitrosourea; ENU) mutagenesis screen generated the Ankrd11^Yod/+^ allele, a missense mutation at a highly conserved residue (E2502K) [26]. The Ankrd11^Yod/+^ or Yoda mouse was reported before the identification of *ANKRD11* mutations in KBG syndrome. The Yoda mice present with craniofacial anomalies that include deformed nasal bones, shortened snouts, and wider skulls and are similar to the craniofacial anomalies seen in KBG syndrome. The Yoda mice also exhibit neuroanatomical perturbations such as neurons misplaced around the lateral ventricles [25] and behavioral abnormalities that are similar to those observed in humans with KBG syndrome [25].

### Clinical management

There are no formal management guidelines published for KBG syndrome. After initial diagnosis, the following have been suggested:

- *Cardiology:* referral to cardiology for echocardiogram
- *Otolaryngology:* palatal assessment and regular hearing tests
  - ○ if otitis media is recurrent, ENT should consider placement of tympanostomy tubes
- *Ophthalmology:* evaluation for myopia, strabismus, and other ocular abnormalities
- *Dentistry:* evaluation for macrodontia, oligodontia and enamel hypoplasia [11]
- *Neurology:* evaluate for abnormal EEG or seizures
  - ○ seizures typically respond to treatment with medication [12]
- *Endocrinology:* emerging evidence suggests that growth hormone may be successful in treating short stature. Referral to endocrinology if pubertal problems are present [27]
- *Urology:* evaluate for undescended testicles
- *Nutrition:* dieticians should be consulted if feeding issues are present [2, 3]
- *Therapies:* for developmental delay and behavior problems. Interventions at school if learning disabilities are present.

### Prognosis

Cognitive: KBG syndrome can be quite variable in regard to the cognitive abilities of affected individuals. There have been no reports of regression. Most children will require support within the classroom, some requiring special education. [3, 12]. Completing a regular high school without additional support appears to be rare; however some reported adults have completed a trade school. More than half had jobs and were self-sufficient [2, 3]. Some were able to live completely independently, while others required some assistance with tasks at home, such as finances. Some affected women have had children and raised them with additional help from a spouse or other family members [2, 3] .

Seizures: Many patients have remission of symptoms after adolescence [12].

Growth: Short stature is common although emerging evidence suggests that growth hormone may be successful in treating short stature [27].

## Conclusions

KBG syndrome is a rare genetic disorder characterized by macrodontia of upper permanent incisors, distinctive craniofacial features, skeletal findings, and developmental delay/intellectual disability with behavioral abnormalities. Awareness of the syndrome will facilitate its clinical diagnosis, which should be confirmed by detecting *ANKRD11* variants. Alternatively, a gene panel or whole exome/genome study would yield a causative *ANKRD11* variant, especially in young children or patients with an atypical presentation. Further studies will help delineating the spectrum of phenotype, details of ANKRD11 function and dysfunction in KBG syndrome, and missing variants either in *ANKRD11* or in additional genes in patients with a clinical diagnosis of KBG syndrome.

## Additional file

Additional file 1: Reported intragenic variants in the *ANKRD11* gene. (XLSX 13 kb)

## Abbreviations

ASD: Atrial septal defect
CdLS: Cornelia de Lange Syndrome
EEG: Electroencephalography
ENT: Ear, Nose and Throat; Otorhinolaryngology
ENU: N-ethyl-N-nitrosourea
IQ: Intelligence quotient
RSS: Russell Silver Syndrome
SD: Standard Deviation
VSD: Ventricular septal defect
